# Peptides to Tackle Leishmaniasis: Current Status and Future Directions

**DOI:** 10.3390/ijms22094400

**Published:** 2021-04-22

**Authors:** Alberto A. Robles-Loaiza, Edgar A. Pinos-Tamayo, Bruno Mendes, Cátia Teixeira, Cláudia Alves, Paula Gomes, José R. Almeida

**Affiliations:** 1 Biomolecules Discovery Group, Universidad Regional Amazónica Ikiam, Tena 150150, Ecuador; alberto.robles@est.ikiam.edu.ec (A.A.R.-L.); edgar.pinos@est.ikiam.edu.ec (E.A.P.-T.); 2 Departamento de Biologia Animal, Instituto de Biologia, Universidade Estadual de Campinas (UNICAMP), Campinas 13083-862, Brazil; bruno000mendes@gmail.com; 3 LAQV-REQUIMTE, Departamento de Química e Bioquímica, Faculdade de Ciências, Universidade do Porto, 4169-007 Porto, Portugal; catia.teixeira@fc.up.pt (C.T.); claudialves05@gmail.com (C.A.); pgomes@fc.up.pt (P.G.)

**Keywords:** amastigote, drug discovery, *Leishmania* spp., peptides, promastigote

## Abstract

Peptide-based drugs are an attractive class of therapeutic agents, recently recognized by the pharmaceutical industry. These molecules are currently being used in the development of innovative therapies for diverse health conditions, including tropical diseases such as leishmaniasis. Despite its socioeconomic influence on public health, leishmaniasis remains long-neglected and categorized as a poverty-related disease, with limited treatment options. Peptides with antileishmanial effects encountered to date are a structurally heterogeneous group, which can be found in different natural sources—amphibians, reptiles, insects, bacteria, marine organisms, mammals, plants, and others—or inspired by natural toxins or proteins. This review details the biochemical and structural characteristics of over one hundred peptides and their potential use as molecular frameworks for the design of antileishmanial drug leads. Additionally, we detail the main chemical modifications or substitutions of amino acid residues carried out in the peptide sequence, and their implications in the development of antileishmanial candidates for clinical trials. Our bibliographic research highlights that the action of leishmanicidal peptides has been evaluated mainly using in vitro assays, with a special emphasis on the promastigote stage. In light of these findings, and considering the advances in the successful application of peptides in leishmaniasis chemotherapy, possible approaches and future directions are discussed here.

## 1. Introduction

Leishmaniasis is a vector-borne protozoan disease, endemic to approximately one hundred countries [1]. It is the second deadliest parasitic disease after malaria, and affects mainly socioeconomically vulnerable communities with complicated access to essential medicines [2]. According to the current epidemiological report from the World Health Organization (WHO), there are nearly 12 million active cases of leishmaniasis. Estimates of the worldwide incidence indicate 1.5–2 million new annual cases, with cutaneous leishmaniasis being two to three times more common than visceral leishmaniasis [3]. The high prevalence of this disease results in 26,000 to 65,000 deaths reported annually, but it is still among the 18 most neglected tropical diseases (NTD) currently recognized by the WHO, posing as a serious public health concern [3].

In humans, leishmaniasis is triggered by at least 20 species or subspecies of endoparasites of the genus *Leishmania* [4]. They are transmitted between hosts through the bite of an infected female sand fly. About a hundred species of these insects belonging to the genera *Phlebotomus* and *Lutzomyia* are known as the main vectors involved in biological transmission [5]. The parasites have a complex life cycle, characterized by the presence of two alternating stages: promastigote and amastigote forms [6]. The promastigote form (invertebrate stage) is responsible for infectivity and is identified by the parasite’s elongated shape and the presence of a long flagellum in its anterior part. In the amastigote form (vertebrate stage), determinant for pathogenesis, *Leishmania* parasites have a spherical shape and present a short flagellum. Amastigotes live, develop, and persist within the host’s mononuclear phagocytic system [7].

The clinical profile resulting from this parasitic infection is categorized into three known disease patterns: cutaneous, mucocutaneous, and visceral leishmaniasis [6,7]. The first form, typified by the formation of skin lesions, is the most common, whereas the last form, distinguished by hepatosplenomegaly, fever, and weight loss, is the most serious version of this disease. In the middle ground, mucocutaneous leishmaniasis is characterized by the damage of oral mucous membranes in the nose, mouth, and throat, which potentiates inflammation and face disfiguration [8].

**Figure 1:**
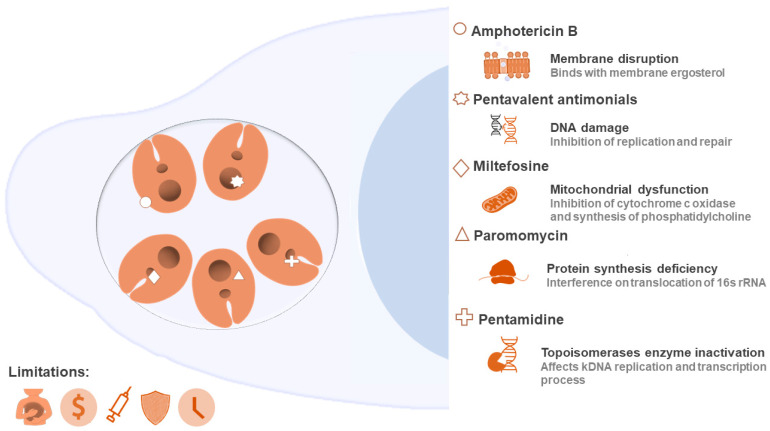
Cellular targets of clinically used drugs against leishmaniasis and their limitations. The targets and action routes are still subject to investigation. This illustration summarizes some of the events underlying the activity of these medicines (membrane disruption, DNA damage, mitochondrial dysfunction, protein synthesis deficiency, and enzyme inhibition). Drugs can act by more than one mechanism, in addition to those detailed here. Several limitations are reported for this traditional chemotherapy, such as toxicity, high cost, difficulty of administration, resistance, and treatment duration, reinforcing the importance of discovering alternative methods.

Leishmaniasis management requires some integrated and multidisciplinary strategies, from insect vector control, enhanced diagnostics, and increased population education, to new therapies with safe and efficient medicines [9]. Due to the absence of a vaccine, drug therapy is the only available alternative. The classical chemotherapy of leishmaniasis is focused on a limited number of high-cost medicines, which are also well known by their side effects [9,10]. In this line, the medical treatments are restricted to the use of pentavalent antimonials, amphotericin B, paromomycin, miltefosine, pentamidine, or combination therapy characterized by the administration of two of the options described above [10]. The clinical antileishmanials act by different cellular and/or molecular strategies, which induce the death of the parasite, but are also widely associated with toxicity and difficulties during administration. Paired with all this, resistance of *Leishmania* spp. parasites to the current conventional chemotherapy is increasing (susceptibility to available drugs is decreasing and is dependent on strains and isolates within the same species) [5,11,12], which is another relevant factor that threatens the adequate treatment of this disease [13]. Figure 1 illustrates the group of Food and Drug Administration (FDA) drugs used to treat a *Leishmania* infection, summarizing their possible cellular targets and main limitations.

Many researchers have highlighted the need to identify new key targets for leishmaniasis treatment, and to develop revolutionary approaches with the help of emerging technologies [10,13]. In recent years, a series of natural and synthetic molecules, including repurposed drugs, have been screened for their leishmanicidal effect. Among these compounds, peptides are a class of molecules that have gained attention, mainly due to their approved clinical use for several other diseases. Ultimately, the increasing interest by the pharmaceutical market in the commercialization of peptide-based drugs emphasizes their utility and clinical success [14,15].

The attractive strengths of therapeutic peptides are rooted in their biocompatibility, versatility, selectivity, low toxicity, tunability, ease of synthesis, and water solubility [16]. Moreover, in the case of antimicrobial peptides (AMP), mainly acting through pathogen membrane destabilization/disruption, they are less likely to select for resistant pathogen strains [17,18]. Due to these peculiarities, peptide-based products have become interesting candidates for the design of an effective and safe treatment for leishmaniasis [19]. The potential of peptides has been reviewed by several publications, highlighting their anticancer [20,21], antifungal [22], and antibacterial properties [23,24], although their activity against protozoan species, mainly *Leishmania* spp., remains largely unexplored. A reduced number of studies have specifically addressed the leishmanicidal effects of peptides, their structural characteristics, achievements, and challenges in this area [14,19,25,26,27]. For this reason, in the present work we have compiled and analyzed the main advances, trends, applications, and characteristics of peptide-based leishmanicidal agents.

Over fifty articles published between 1992 and 2020, describing peptides evaluated against *Leishmania* infection, were reviewed. The primary structure (amino acid sequence) of peptides with antileishmanial activity reported in the literature was collected and used to elaborate a database (Supplementary Material, repository available since 25 March 2021 from: https://github.com/albert-robles1101/Leishmania-database, accessed on 18 April 2021). Other relevant information, such as IC_50_, species, and parasite form were also obtained from these references. In addition, with the help of the ‘Peptides’ package of the software R, some physicochemical parameters were calculated in order to gain more insight into the structure and leishmanicidal potential of these peptides. Our database contains nearly 300 entries, taking into account the characteristics analyzed—so the same peptide sequence can have two or more entries when tested against more than one species or more than one stage of parasite development.

## 2. Peptides Targeting *Leishmania* spp.: Current Progress

To date, more than 140 unique peptides have been identified which affect the parasitic growth of at least one *Leishmania* species, either at the promastigote stage, amastigote stage, or both [28]. The Parapep repository (http://crdd.osdd.net/raghava/parapep/, accessed on 20 February 2021), focused on antiparasitic peptides, currently provides detailed information on about 80 unique sequences proven to be active against *Leishmania* (reviewed in March 2021) [29]. Therefore, this review considerably expands the number of known peptides targeting leishmaniasis.

**Figure 2:**
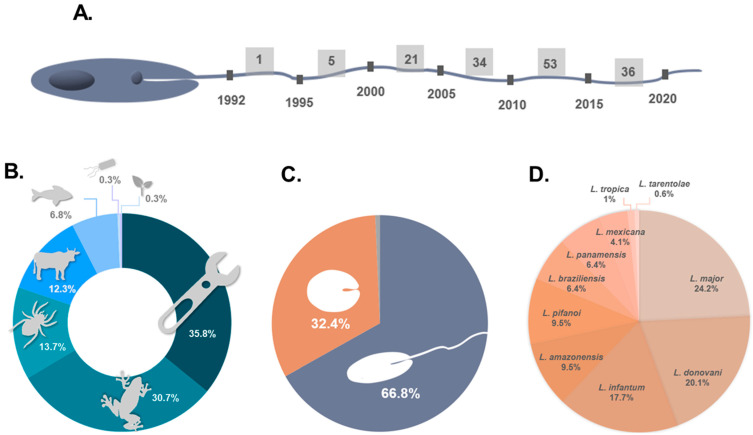
Current overview of peptides with leishmanicidal effects. (**A**) Timeline of investigation addressing peptides with anti-promastigote and/or anti-amastigote effects. (**B**) Distribution of peptides into 7 clusters, according to natural or engineered origin. The percentages were calculated from 150 unique peptides described in the literature. (**C**) Parasite life forms used in the screening of antileishmanial activity of peptides (293 total entries). (**D**) Comparison of the species of *Leishmania* used in the studies.

The first antileishmanial peptide ever found was reported in 1992 by Hernandez and collaborators [30]. It was dermaseptin, a 34-amino acid residue peptide isolated from frog skin secretions, active against promastigotes of *L. mexicana*. Since then, many research groups have been dedicated to the discovery and structural elucidation of novel peptides isolated from different organisms [31]; additionally, researchers have also explored peptide engineering, which generated useful guidelines for the rational design of antileishmanial chemical entities [32,33]. As a result, both approaches have contributed to expanding the library of peptide structures available for the battle against leishmaniasis. In line with this, Figure 2 outlines the state of peptide research targeted at *Leishmania* protozoans.

Analysis of several publications highlighted a substantial increase in the amount of peptide data available throughout each decade (Figure 2A). Between 1991 and 2000, only half a dozen peptides with activity against at least one species of *Leishmania* had been characterized. Between 2001–2010, this number dramatically increased, with the discovery of over fifty new peptides, and even more in the last decade (2011–2020), with nearly ninety new peptides presenting antileishmanial properties being reported. The most relevant years in terms of the discovery of these bioactive molecules were 2008, 2011, 2012, and 2016. Therefore, in general, the last decade stands out, probably thanks to the advances in omics technologies, particularly proteomics and peptidomics, which contributed to the knowledge of both peptides and structural models for *Leishmania*, a trend that has also been observed for AMPs [34] and anticancer peptides (ACPs) [35]. Based on this preamble and future technological advances, new amino acid sequences and/or antileishmanial leads are expected to appear in the coming years.

Synthetic peptides can be replicas of the sequences available in nature [36] or improved versions thereof—shorter [37], hybrid [38], or wider-scope [39], or versions with chemical modifications [40] or amino acid substitutions [39,41]. In addition, some synthetic peptides are obtained by mimicking specific regions of proteins or toxins [42,43]. Considering this, the natural antileishmanial peptides reported to date and their synthetic versions (peptides obtained by chemical synthesis having the exact primary structure found in nature) can be categorized into six clusters, according to their source: amphibians, bacteria, marine organisms, invertebrates, mammals, and plants. A seventh cluster can be added for peptides bearing non-natural chemical modifications, encompassing amino acid substitutions, or derived from a protein sequence. Figure 2B shows the distribution of the peptides according to the groups described above. Even though there is still structural diversity within each group, the most outstanding source of antileishmanial peptides is the amphibian cluster (30.72%). Skin frog secretions have been described as a therapeutically relevant library of components, including peptides widely capable of inducing the death of micro- and macroscopic pathogens [44,45,46,47], and with demonstrated activity against both life cycle stages of *Leishmania* [36,48,49,50]. Figure 2B also shows the relevance of structure–function and peptide design studies (7th cluster), as well as the modifications carried out to functionalize peptides for clinically useful applications. In total, 35.84% of peptides in our database are chemically modified, either derived from (or inspired by) proteins or containing substitutions of amino acid residues. Different strategies have been employed to obtain more efficient and less toxic versions of peptides. For example, CA (1–8) M (1–18) (IC_50_ = 1.3 µM) and D-CA (1–8) M (1–18) (IC_50_ = 0.4 µM) are hybrid peptides of cecropin A (IC_50_ > 50 µM) and melittin (IC_50_ = 0.3 µM), which act against *Leishmania donovani* [38]. Likewise, F5W–magainin 2, a peptide designed to reduce the effective dose of lysis in human erythrocytes (IC_50_ > 1000 µM over these human cells), comes from the conjugation of highly toxic versions MG–H1 (ED_50_ = 2.9 µM) and MG–H2 (ED_50_ = 16 µM) [41]. This combination of two peptide sequences significantly decreased the damage to red blood cells. Therefore, as exemplified, the construction of artificial hybrid peptides is a useful approach for obtaining more effective and/or less toxic peptides [38,41].

Regarding the parasite’s life form used for the screening of peptides’ activity, of the nearly 300 entries analyzed, 66.89% correspond to the promastigote stage, 32.42% correspond to the amastigote stage, and 0.68% do not mention the parasite stage considered in the investigation (Figure 2C). Therefore, these data show that the scientific community has mostly evaluated the anti-promastigote effects rather than anti-amastigote activity of *Leishmania*. Future investigations could either contribute to filling this gap by specifically targeting the clinically relevant amastigote stage and considering the ensuing translation into therapeutic modalities, or taking advantage of the large amount of information on the activity of peptides against the promastigote stage to develop a useful control of the transmission by transgenesis or para-transgenesis strategies [51,52].

Figure 2D illustrates the different *Leishmania* species against which peptides have been tested. Although there are a total of 20 known species of *Leishmania* [53], only 10 species have been assayed in vitro, according to the literature. *L. major* (24.23%), *L. donovani* (20.14%), and *L. infantum* (17.75%) are the main targets of studies involving peptides. Regarding their clinical manifestation, *L. major* is one of the causative agents of localized cutaneous leishmaniasis, whereas *L. donovani* and *L. infantum* are responsible for visceral leishmaniasis or black fever [28]. In general, at least 60% of in vitro experiments with leishmanicidal peptides have aimed at finding an alternative to current treatments for localized cutaneous or visceral leishmaniasis. On the other hand, around 40% of in vitro experiments looked for solutions that attack the causative agent of diffuse cutaneous and mucocutaneous leishmaniasis.

**Figure 3:**
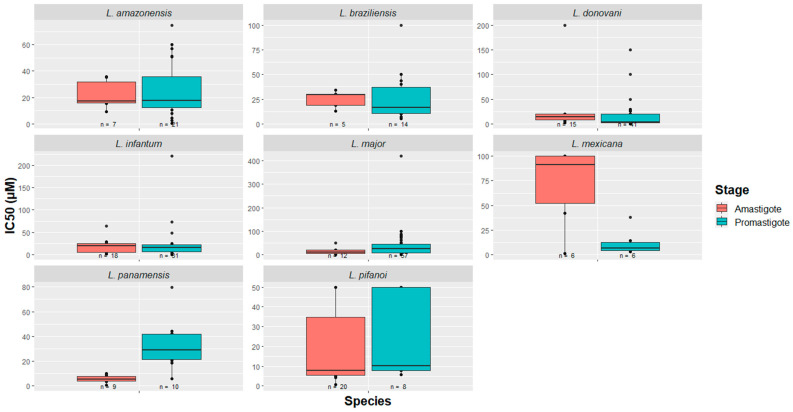
Peptides as effective agents against *Leishmania* spp. Potential of these molecules for leishmaniasis drug therapy are confirmed by the IC_50_ values described in the literature (in vitro studies). The majority of peptides act in the micromolar range.

Of the ten *Leishmania* species studied, eight of them presented more than three data entries in their respective promastigote/amastigote stages. Taking this into account, a box plot was constructed, as presented in Figure 3. Since *L. tropica* promastigotes have only been tested with Temporin Shd (IC_50_ = 13.9 µM) [54], and since *L. tarentolae* has only been used for the screening of the potential of KDEL peptide in both stages (with unknown IC_50_ values) [55], these species were not included in the box plot. As for the remaining species, the activity ranges of the peptides vary between small values, on the order of nM, to values greater than 100 µM. Examples of the most effective peptides are 27RP (IC_50_ = 0.017 pM in *L. donovani*) [56], indolicidin (IC_50_ = 35 pM in *L. donovani*) [56], and RI–1018–NH_2_ (IC_50_ = 0.6 µM in *L. major*) [26]. The maximum entry number (*n* value) reported per species in the promastigote stage is 57, corresponding to *L. major*, whereas for the amastigote stage, the maximum number of entries per species is 20, for *L. pifanoi*. These observations open a precedent for future peptide research against *Leishmania* protozoans by providing a broad picture of how much the species have been studied in each of the stages, as well as the in vitro potency of the evaluated molecules.

In general, the attractiveness of peptides lies mainly on their biocompatibility, structural versatility, and low probability of inducing the selection of resistant strains when compared to traditional drugs. This is in addition to the other features, previously mentioned in the introduction, which make peptides rather interesting for drug development and translational medicine, especially within scope of the so-called “post-antibiotic era” [57,58,59].

**Figure 4:**
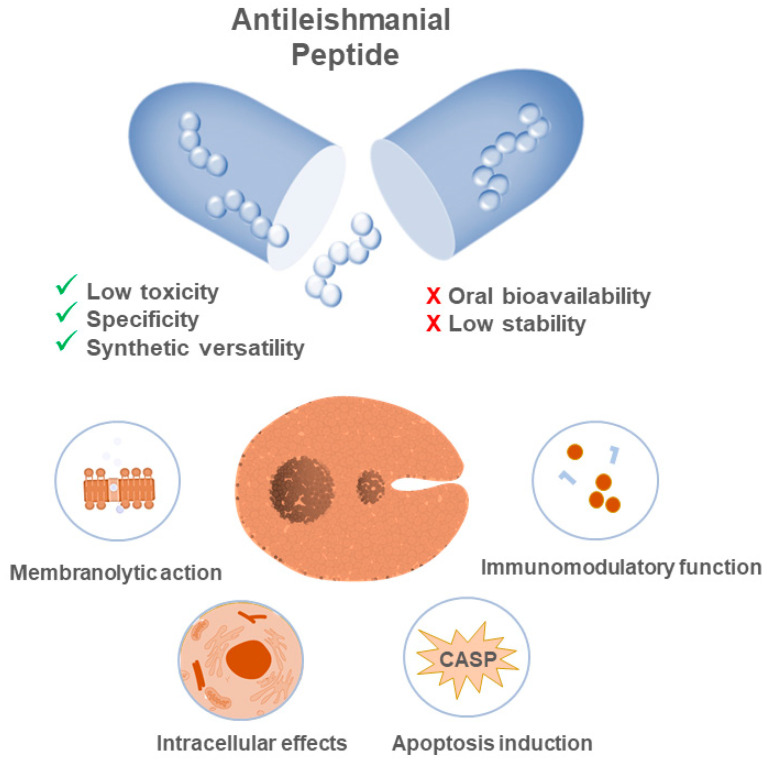
Peptides as new antileishmanial drugs: mechanisms behind their effectiveness, benefits, and challenges for clinical applications. The activities of peptides occur due to different biochemical processes, among which the disruption of the membrane, the interaction with intracellular targets, and the modulation of the immune response of the host stand out. The use of molecules of this nature has several advantages, such as the fact that they are target-directed, easy to modify, and have low toxicity for the host. The most common challenges when using these molecules lie in achieving structural stability and bioavailability for in vivo applications.

The antileishmanial activity of peptides is thought to occur via some of the following biochemical processes—membrane disruption of the parasite, either promastigote or amastigote; activation of apoptotic signals; or via modulation of intracellular targets or of immunomodulatory responses (Figure 4). In order to illustrate these events, Zhid et al. [39] demonstrated that Temporin SHa and Temporin [K3] SHa are capable of forming blisters in the promastigote membrane, causing the loss of its integrity and consequential cell death. This disruption is based on electrostatic interactions between the cationic peptides and the negatively charged glycocalyx of the membrane, which contains lipophosphoglycan (LPG) and proteophosphoglycan (PPG). This event culminates in the prompt formation of pores dependent on the concentration of peptides. In another example, Lynn and collaborators [60] analyzed the effect of BMAP–28, a peptide that promoted the damage of the parasite’s vacuoles and membrane, resulting in late cellular apoptosis as a direct consequence of DNA degradation. Pérez et al. [61] demonstrated the immunomodulatory properties of andropin and cecropin A, short peptides that inhibited intracellular amastigote stage parasitic growth, mediated by interactions established with crippled phagocytes.

The main parasitic stage on which most peptides have been tested is the promastigote stage, which already poses a limitation. In addition, the peptide’s specificity and non-toxicity to the host cell represents an additional barrier. Thus, only a very small fraction of leishmanicidal peptides have reached in vivo trials, and none has advanced to clinical trials thus far. New insights into the potential of these molecules in vivo are key to assessing the limitations related to their nature and to improving the design of strategies that could guarantee their clinical success [19].

One of the first studies evaluating their activity in vivo was carried out by Alberola et al. (2004) [62]. The acylated synthetic antimicrobial peptide Oct–CA (1–7)M (2–9) was employed as an agent for the treatment of dogs diagnosed with leishmaniasis caused by *L. infantum*. All animals received 5 mg of the peptide by intravenous injection. Hematological and biochemical tests showed a decrease in parasitemia (the number of parasites in the blood). Furthermore, the animals did not show any side effects even after 6 months post-treatment, confirming the efficacy and security of the peptide. However, its mode of action has not been explained in detail.

Mukherjee, Ukil and Das [63], working with the cystatin peptide, evaluated the response mechanisms developed by macrophages to an established infection with *L. major*. The experimental design included the injection of various peptide doses for 4 days in the tail vein of BALB/c mice, and new analyses 45 days after the last injection. The treatment regimen modulated the levels of signal molecules and triggered mechanisms that even protected the model organism from reinfection, which supports cystatin as a promising model for antileishmanial immunotherapies.

Erfe et al. [64] focused on the activity of peptides RP–1 and AA–RP–1 against infection by *L. infantum chagasi*. Mice were treated with seven intravenous doses of 0.25 mg of the peptides and were euthanized on day 20. Both the spleen and liver extracted from the mice showed a low parasite load compared to the negative control, which suggests the effectiveness of both peptides in vivo against visceral leishmaniasis.

The work of Campos-Salinas et al. [65] presented the action of urocortin II in vivo. This peptide is produced in the human body as part of the innate immune system; therefore, it has a highly conserved primary structure with anti-pathogenic activity. The evaluation, carried out 7 weeks after infection, demonstrated that this peptide can significantly reduce the lesions and necrosis generated by *L. major* infection, both in the epidermis as well as in the cartilage of injected footpads. In a follow-up work by the same group, the in vivo action of the VIP peptide and its analogues was demonstrated in mice infected with *L. major*. VIP (1.5 nmol/mouse) was injected for 7 weeks, 3 times a week, in the same paw in which the promastigotes were injected. The VIP derivatives reduced the parasite load, the cutaneous lesions, and the spread of the infection to visceral organs [66].

The study on the effectiveness of human neutrophil peptide 1 (HNP-1) carried out by Abdossamadi et al. [67] is one of the most recent examples of the in vivo assessment of antileishmanial peptides. In this case, BALB/c mice were infected with promastigotes of *L. major*, then divided into five groups (15 per group) and each was subjected to a different route of peptide administration at different doses and intervals. A significant reduction in parasitemia in specific tissues and an increase in the expression of signal molecules favorable to the control of disease progression were reported.

Several peptides with leishmanicidal action are also key components of the innate immune system of their producing organisms. Consequently, they can modulate the host’s immune system through the production of signal molecules and the regulation of gene expression, in order to achieve more effective parasite inhibition [68]. Some immunotherapy approaches based on AMPs have been slightly explored [14], but only a small number of immunomodulatory peptides have been evaluated in vivo on *Leishmania* spp. One example is that of the HNP-1 peptide, mentioned above, which was further studied by Abdossamadi et al. with a focus on immunotherapy [69]. In this work, *L. tarentolae* was modified to encode the HNP-1 peptide sequence, taking advantage of the fact that *L. tarentolae* is not pathogenic and has long been considered an important model for the development of vaccines against leishmaniasis [14]. This species was found to be a highly effective HNP-1 secretor; hence, BALB/c mice infected with *L. major* were injected with peptide-secreting *L. tarentolae* parasites (2 × 10^5^ cells/50 μL, once a week for 3 weeks) and treatment outcomes were analyzed by real-time PCR. This revealed that the *L. major* load in the mice lymph nodes decreased significantly compared to control groups.

The synergistic action of peptides or of peptide–drug combinations has been extensively investigated for antibacterial and carcinogenic effects and has triggered interest in covalent conjugates between different bioactive building blocks. New conjugates have been obtained through the synthesis of amino acid sequences embedding peptide 1 + peptide 2 (aka chimeric peptides) or peptide + drug in a single molecular construct, using different spacers and conjugation chemistries. This type of strategy has also been tested against *Leishmania*, such as in, e.g., the study carried out by Luque-Ortega et al. [70]. These authors linked the cell penetrating peptide Tat (48–60) to miltefosine, one of the most effective oral drugs against leishmaniasis (especially visceral), against which parasite resistance has been emerging. The conjugation of both moieties was carried out through either a disulfide or a thioether bond, and in both cases inhibition of *L. donovani* was achieved, validating the potential of this approach.

In another study, the same cell-penetrating peptide was conjugated to paramomycin, an antibiotic that has been used as alternative anti-leishmanial therapy, but which possesses a low capacity to disrupt membranes. In this work, Defaus et al. [71] applied a chemoselective conjugation strategy that linked the side chain carboxyl of a C-terminal Asp residue from the peptide to the aminomethyl group from the antibiotic, hence conserving the key functional groups for the activity of both molecules. The conjugate thus obtained was active against promastigotes of *L. donovani*, and the internalization in the parasites was confirmed by confocal fluorescence microscopy. Hence, conjugation of different bioactive moieties can convey synergistic effects to provide an overall improved anti-leishmanial profile.

## 3. A Snapshot of Antileishmanial Peptides from the Literature

Efforts to guide therapeutic peptide-based research have privileged health conditions other than leishmaniasis, e.g., cancer and bacterial, viral, or fungal infections. This can be corroborated by analyzing the entries on different peptide databases. One example is ‘The Antimicrobial Peptide Database’ [72], which has 3257 search entries based on the peptide’s targeted action (antibacterial, antiparasitic, anticancer). The total number of entries for antiparasitic peptides is 138, and of these, only 36 have been reported to be active against *Leishmania* parasites. Most of the antileishmanial peptides covered by this review are found in this database. ‘Parapep’, another relevant database, has 137 entries of *Leishmania*-directed peptides [29]. Both databases have substantially more information on peptides directed towards bacteria or malaria parasites. Furthermore, the absence of antileishmanial peptides in clinical settings is evident from the evaluation of ‘THPdb’, a database of FDA-approved peptides [73] that does not include any peptide-based drugs for leishmaniasis therapy.

**Figure 5:**
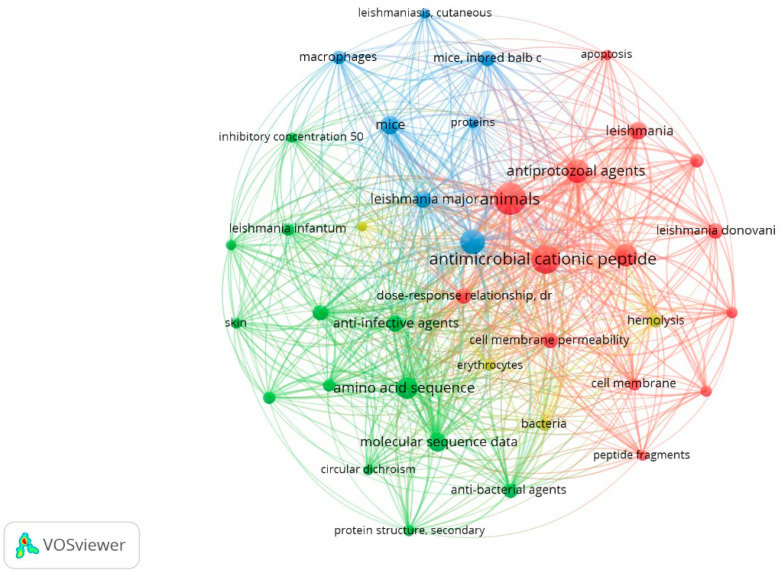
Bibliometric VOSviewer map of the key MeSH terms including *Leishmania* species, experimental techniques, and sources of the most frequently occurring peptides. This network was built using co-occurrence analysis and the full counting method. The size of the circles is proportional to the level of occurrence of the key terms in the articles. Co-occurrence is the determinant for the proximity of the circles. Out of 267 keywords found, only 38 are displayed and the maximum occurrence level is 5. The total graph has 554 links, and the total link strength is 1750.

In view of the above, and to expand our understanding on the current state of antileishmanial peptide research, a bibliometric map based on VOSviewer Software and medical subject headings (MeSHs) was constructed, using articles describing the antileishmanial activity of peptides (Figure 5). This tool for research data management is usually applied when a more comprehensive view of a bibliographic network is needed.

Four clusters, denoted in Figure 5 by the colors blue, green, red, and yellow, represent the network connection of the MeSH terms retrieved from the evaluated articles. The results shown here coincided with the analysis carried out with the database entries used in this article. The three main species used for in vitro assays are present in three of the identified clusters—*L. infantum*, *L. major*, and *L. donovani*, colored in blue, green, and red, respectively. The proximity of the MeSH terms indicates more frequent co-occurrence. The blue cluster gathers aspects associated mainly with in vivo action of the peptides and the experimental laboratory mice employed in translational research. Furthermore, the green cluster highlights keywords related to the structural characterization of these anti-infective agents. The set of red items brings together the most common mechanism of action of cationic peptides and their molecular target. The main yellow descriptors include the cell type and hemolytic assay widely used to assess the toxicity of peptides. Despite the typical features of each cluster, a highly connected network is formed, revealing the integration of different MeSH descriptors and multidisciplinary approaches in the field of antileishmanial peptide research.

Peptide cationic character and animal origin are two characteristics highlighted in the bibliometric VOSviewer map. These data are corroborated by the peptides’ charge, determined in silico and available in our database—approximately 94% of the peptides that were able to obtain their required biophysical properties had a positive charge (*n* = 111). A net positive charge has been widely indicated as a determining factor for the leishmanicidal activity of many peptides [74,75,76], and as previously depicted in Figure 2, peptides of animal origin have gained notable attention for antileishmanial drug development in the last two decades. In this connection, amphibians have been the most studied group.

In each cluster, the experimental methods used are also denoted. The map suggests great efforts aimed at elucidating the structure of the peptides, with special emphasis on the secondary structure characterized by circular dichroism (green descriptor). When it comes to toxicity, assays using macrophages (blue cluster) and erythrocytes (hemolysis, yellow descriptor) are the most common. Permeability tests (red item) are also often considered in order to understand the mode of action of the peptides, which is in many cases linked to their interaction with *Leishmania* membrane components, mainly against promastigotes. For this reason, membrane permeability and cell membrane are also keywords that are clearly visible on this map.

Another interesting observation on citation trends is the peptides’ multifunctionality or versatility against different infectious agents. Just as drug repurposing has been highly valuable as a short pathway for drug discovery, the repositioning or recycling of peptides has also been a promising route used to identify new peptides against *Leishmania* spp. Many peptides currently recognized for their ability to combat *Leishmania* were primarily evaluated as anticancer or antibacterial agents. An example of this peptide recycling is reported in a study by Pérez-Cordero and collaborators [61]. These authors worked with andropin, cecropin A, cecropin B, cecropin P1, dermaseptin, melittin, tachyplesin, Pr-1, Pr-2, and Pr-3, all of which were initially considered antibacterial agents. In this same scenario, the kahalalide F depsipeptide and its analogues are another interesting example, which at first instance were recognized for their antitumor activity directed at prostate cancer. Later, these small peptides demonstrated promising features for leishmaniasis treatment [77].

Several studies have explored the in vivo antileishmanial properties of peptides [42,48,55,60,64,78,79,80,81]. The preferred animal model for this type of analysis was the BALB/c mouse, as corroborated by the bibliometric map. All investigations using this model have confirmed the applicability and maintenance of the leishmanicidal action of the selected peptides in vivo.

## 4. Understanding the Primary Structure and Composition of Leishmanicidal Peptides

Peptides targeting protozoans are heterogeneous molecules with a high structural and functional diversity, which underlies their characteristic wide-spectrum activity against different cell types, such as bacterial, fungal, parasitic and tumoral [68,82,83]. Cationicity and amphipathicity of antimicrobial (including antiprotozoal) peptides have been constantly reported as key features for their activity and proposed mechanisms of action, which involve microbial membrane destabilization. Indeed, the importance of the initial electrostatic interactions between antiprotozoal peptides and the predominantly negative membranes of target cells has been widely reported, as this interaction promotes either the destruction or permeabilization of parasite membranes, causing their death due to either the loss of vital intracellular contents or the impairment of normal organelle functioning [84].

**Figure 6:**
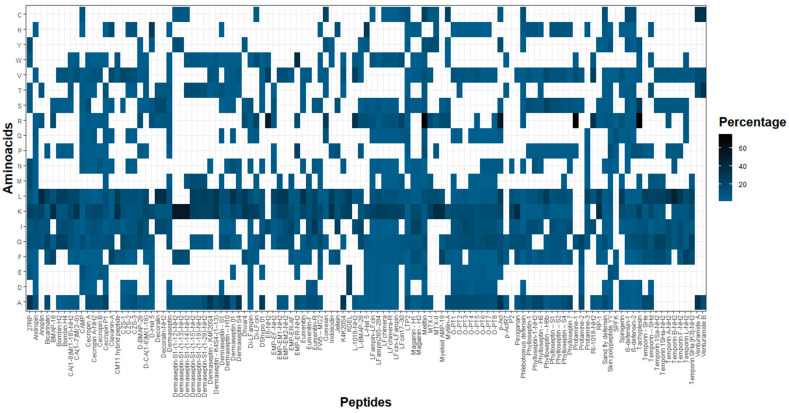
Heat map of natural amino acids in leishmanicidal peptides. Lysine (K) and leucine (L) are the most commonly found.

In connection with the above, it is not surprising that most of the antileishmanial peptides herein revised are characterized by high contents of basic (positively charged) amino acids. Of the three positively charged natural amino acids—histidine (H), arginine (R) and lysine (K)—the latter is the most common, as shown by the heat map in Figure 6. Examples of lysine-rich antileishmanial peptides include Bmap–18, which displays an IC_50_ = 12.81 µM against *L. donovani* promastigotes [79]; and RP–1, with IC_50_ values of 50 µM for *L. braziliensis* and *L. chagasi*, and of 100 µM for *L. major*, in their promastigote forms [64]. Another example is that of the 13-mer peptide p–Acl, which possesses seven Lys residues and displays IC_50_ values in the 51 to 220 µM range for promastigotes, and in the 25 to 50 µM range for amastigotes of different strains of *L. amazonensis* and *L. major* parasites [42].

Interestingly, replacing lysine residues by arginines, despite conserving the peptide’s net charge, often leads to different bioactivity and membrane interaction properties. Owing to its side chain guanidinium group, as compared to the ammonium in lysine, arginine residues have a greater capacity to interact with cell membranes, and therefore induce modifications that more strongly compromise parasite viability [85]. Interestingly, peptides in our database that are characterized by a high percentage (≥60%) of a given amino acid often have arginine as the preferred one. Peptides derived from protamines (Pr–1, Pr–2, and Pr–3) belong to this group, and when evaluated against promastigotes of *L. panamensis*, Pr–3 presented the highest inhibitory activity (IC_50_ = 5.74 µM) [61]. Another example is the E–6 peptide, which was evaluated on promastigotes and amastigotes of *L. major* and *L. donovani*, yielding an IC_50_ = 20 µM for both species and developmental stages [26]. These results suggest a greater antileishmanial potency for arginine- as compared to lysine-rich peptides, though a meaningful comparison can only be made by replacing lysine by arginine residues in a given sequence.

This was done by Mendes et al., who developed the aforementioned 13-mer peptide p–Acl, as well as its all-arginine analogue, p–Ac1R7. The latter peptide had stronger activity than the all-lysine one against both promastigotes and amastigotes of the different species/strains of *Leishmania* parasites tested (27 to 71 µM for promastigotes; 16 to 18 µM for amastigotes); moreover, p–Ac1R7 displayed a faster parasite membrane-permeation action than p–Ac1 [42]. These observations are in line with studies involving antimicrobial and anticancer peptides that have shown a greater capacity to penetrate cells when having a high density of arginine residues, as compared to analogs of the same length containing lysines instead [85,86].

Another abundant amino acid in antileishmanial peptides is leucine (L), a nonpolar amino acid with an aliphatic side chain. In nature, peptides containing leucine-rich motifs are common, mainly among antimicrobial peptides. This and other hydrophobic amino acids play a fundamental role in the amphipathicity of antimicrobial peptides, and in their ability to bind and destabilize the membranes of microorganisms [87]. Some examples of leucine-rich peptides (>40%) include anoplin [88] (IC_50_ = 87 µM on *L. major* promastigotes) and decoralin [89] (IC_50_ values = 72 and 11 µM on *L. major* promastigotes, for the native and the C-terminally amidated peptides, respectively). Temporin B, which is also included within this group, was evaluated on *L. pifanoi* and *L. donovani*, the latter being the most susceptible in promastigotes and *L. pifanoi* in amastigotes (IC_50_ = 8.6 µM and 5 µM, respectively) [36].

Additionally, studies have associated the resistance to salt gradients and proteolytic excision with the presence of leucine residues in antimicrobial peptides [29]. These features are equally relevant for antiamastigote and antipromastigote action and are particularly important for the in vivo efficacy of the peptides. However, it is important to balance the pros and cons of a high content in leucine, since many leucine-rich peptides have shown high toxicity, which limits their clinical application [30,31,90,91].

In addition to leucine, seven other hydrophobic amino acids (alanine, isoleucine, valine, proline, phenylalanine, tryptophan, and methionine) have a noticeable presence in several of the peptides presented in Figure 6. The hydrophobicity resulting from the presence of these residues has been pointed out as a special factor for the selectivity and potency of these bioactive molecules. An analysis of the sequence of antimicrobial peptides determined that these amino acids constitute 50% of their sequences [92,93]. Some examples of leishmanicidal peptides that possess or exceed this percentage of hydrophobic amino acids are dermaseptin–K4S4–(1–13) (53.84%) [94], magainin H–1 (56.52%) [41], L–1018 (58.33%) [26], RP–1 (72.22%) [64] and cruzioseptin–1 (76.19%) [46].

Negatively charged amino acids are not abundant in antimicrobial peptides, including those with antileishmanial action. Of all the sequences reviewed in this work, <30% present at least one of the two negatively charged amino acids—aspartic (D) and glutamic (E) acids. One of the most prominent examples of this group, given its nature, is the short peptide named after its amino acid sequence, KDEL, which presents a higher amount of negative amino acids (50%). This peptide is active against both life cycle stages of *L. tarentolae* (IC_50_ not specified) [55]. Nevertheless, as generally observed for antimicrobial peptides, these negatively charged amino acids do not seem to contribute significantly to the peptides’ activity against *Leishmania*, and may even hamper the initial event of interaction with the parasite’s membrane.

Each peptide is unique regardless of its similarities with other peptides. The combination of amino acids is the result of innumerable evolutionary processes, which were established based on the needs of their producing organism. All these changes imply gains or losses in molecular interactions, which give rise to new properties or characteristics to the peptide sequence that also influence secondary structure and biological activities [59,95]. Several computational tools have been developed and are available to assist in the design or in silico evaluation of anticancer or antimicrobial peptides. Some examples include the Antimicrobial Sequence Scanning System (AMPA; http://tcoffee.crg.cat/apps/ampa/do, accessed on 18 April 2021), Pepdraw (https://www.pepdraw.com/, accessed on 18 April 2021), PEP-FOLD (https://bioserv.rpbs.univ-paris-diderot.fr/services/PEP-FOLD/, accessed on 18 April 2021), the database of antimicrobial activity and structure of peptides (DBAASP; https://dbaasp.org/, accessed on 18 April 2021), and CancerPPD (http://crdd.osdd.net/raghava/cancerppd/, accessed on 18 April 2021). However, tools of this kind for the specific prediction or design of peptides targeting *Leishmania* parasites remain unavailable.

## 5. Deeper Insights into the Physicochemical Properties of Antileishmanial Peptides

**Figure 7:**
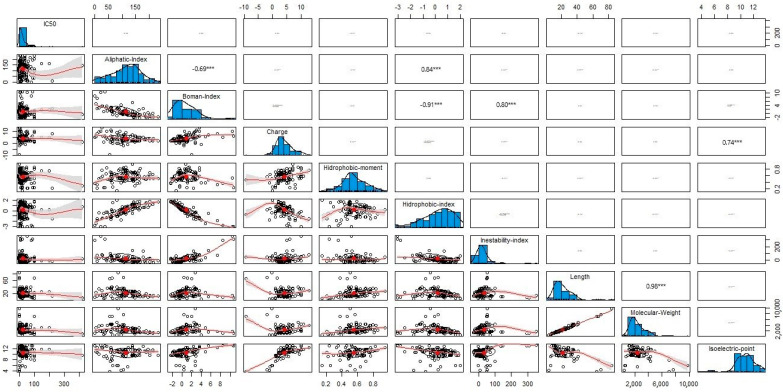
Distribution and trends of the biophysical descriptors of leishmanicidal peptides. In general, the biophysical parameters explored have a non-normal Gaussian distribution. The relation between biophysical descriptors are |r| ≥ 0.65: aliphatic index–Boman index (r = −0.69), aliphatic index–hydrophobic index (r = 0.84); Boman index–hydrophobic index (r = −0.91), Boman index—instability index (r = 0.80), charge–isoelectric point (r = 0.74), and length–molecular weight (r = 0.98).

Unravelling not only structural, but also physicochemical patterns of leishmanicidal peptides is of the utmost importance in the prediction, identification, and design of peptide-based antileishmanial agents. Hence, in this study, biophysical descriptors—namely, the aliphatic index, Boman index, charge, hydrophobic moment, hydrophobic index, instability index, length, molecular weight, and isoelectric point—were calculated in silico using the “Peptides” package of R software [96]. Figure 7 summarizes the main findings of this analysis.

The values of the physicochemical parameters of the leishmanicidal peptides are within the following ranges: 114.63 ± 53.30 (aliphatic index), 0.87 ± 2.64 (Boman index), 4.07 ± 2.99 (charge), 0.56 ± 0.15 (hydrophobic moment), 0.19 ± 1.21 (hydrophobic index), 31.99 ± 67.58 (instability index), 21.48 ± 11.22 (length), 2412.66 ± 1227.57 (molecular weight), and 10.58 ± 1.75 (isoelectric point) (Supplementary Material, Table S1). A non-Gaussian distribution was observed for each physicochemical property, as corroborated by the Shapiro–Wilk robust normality test. Figure 7 also shows the correlations between the physicochemical parameters of the peptides, of which the following should be highlighted for having a Pearson correlation coefficient |r| ≥ 0.65: aliphatic index–Boman index (r = −0.69), aliphatic index–hydrophobic index (r = 0.84); Boman index–hydrophobic index (r = −0.91), Boman index–instability index (r = 0.80), charge–isoelectric point (r = 0.74), and length–molecular weight (r = 0.98).

A statistical model correlating leishmanicidal activity (IC_50_ values) with any of the physicochemical properties mentioned above is yet to be established. Previous reports, such as those by Lee et al. [97], Wang et al. [72], and Waghu et al. [98], highlighted the relevance of certain biophysical parameters for the action of antimicrobial peptides. However these should not be regarded as extensible to antileishmanial peptides, not only because the number of known leishmanicidal peptides is still small, but also because not all reported antimicrobial peptides possess antileishmanial properties. In fact, when comparing the number of peptides from Lee’s study with the number of peptides from a large-scale investigation such as that of Wang et al. [99], ADAM’s validated list of antimicrobial peptides [97] contains only 3.52% of Wang’s corresponding data. Still, the complete existing background on antimicrobial peptides could guide future research focused on the development and validation of computational models for leishmanicidal peptides.

## 6. Perspectives and Directions for Future Research on Antileishmanial Peptides

Several mechanisms and structural parameters have been proposed as underlying the action of peptides against *Leishmania* parasites, but they are mostly based on what is known about antimicrobial peptides in general, and hence lack specificity. A better understanding of the mechanisms and structural features that are determinant for antileishmanial peptides is essential for the future design and optimization of drugs based on these molecules [22].

As shown by the cited examples, the main *Leishmania* species used in the assessment of antileishmanial peptides have been *L. major* and *L. infantum*. Although to date no specific reason has been clearly advanced for this choice, the focus on *L. infantum* is likely due to the fact thatbecause this is one of the main species that causes visceral leishmaniasis, which, according to PAHO (Pan American Health Organization) [100], is fatal to more than 90% of untreated patients. In turn, *L. major* is one of the main causative agents of cutaneous leishmaniasis in the Old World [101]. Wide interspecies differences in terms of structure, immune system evasion mechanism, and infection severity [102] add complexity to the understanding of the different variants of leishmaniasis. Most of the leishmanicidal peptides herein addressed were collected from studies that were carried out in different countries, where the most representative species (epidemiologically reported or identified in the territory) were likely the preferred ones. To unravel this, an associative region-versus-species analysis like the one conducted by Boussoffara et al. in Tunisia [103] should be performed.

Considering the large number of promising antileishmanial peptides that have been identified in vitro, the corresponding in vivo assessment is substantially underexplored. In fact, the in vivo assessment of bioactive peptides remains a challenging field of research, given the well-known bioavailability issues of peptides. Typically, peptides have a short life span in vivo, given their rapid elimination and susceptibility to proteases [104]. To overcome these drawbacks, different strategies have been proposed, encompassing, e.g., the use of non-canonical amino acids (D-amino acids, N-methylated amino acids, and others); C-terminal amidation; N-terminal acetylation; cyclization; encapsulation in nanocarriers; conjugation with biomolecules, such as lipids, sugars, other peptides, or others; and the design and synthesis of peptidomimetics [17]. However, none these specific strategies can be regarded as a universal tool, as modes of action and targets vary from one peptide to another [105].

Notwithstanding, a few steps have already been taken to improve the bioavailability of antileishmanial peptides. For instance, L–1018 is an antileishmanial peptide composed of D-amino acids developed by Marr et al. [26] that stood out from a group of four peptides by exhibiting the lowest IC_50_ value (0.6 µM) against promastigotes of *L. major*. In another work, Ruiz-Santaquiteria et al. [106] designed a series of cyclic peptides from a linear prototype to act as both as dimerizing agents and inhibitors of *L. infantum* trypanothione reductase. Results were quite promising, and the cyclic peptides were further conjugated to cell-penetrating peptides, which improved antiparasitic activity, affording IC_50_ values in the 3.5 to 6.4 µM range for promastigotes, and in the 1.5 to 10.4 µM range for amastigotes. Still, considering the scarcity of in vivo studies involving antileishmanial peptides, future research should focus on both the evaluation and optimization of the action of these peptides in animal models of leishmaniasis.

Another major challenge in advancing peptides for the treatment of leishmaniasis concerns the intracellular location of the clinically relevant stage, i.e., amastigotes. Given their residence inside macrophages and their distinct membrane composition, amastigotes represent an additional complication for the design of effective treatment strategies [68,84,107]. To inhibit amastigote development, the peptide must have the ability to penetrate both the macrophage and the parasite membranes. Additionally, the interaction with host cells must not represent the damage or destruction thereof [49,87]. Despite many of the peptides reviewed here having succeeded in eliminating amastigotes within the host cells, most reports reveal a more effective peptide action against promastigotes than against amastigotes. This aspect can be explored in two ways—on the one hand, investing in improving peptide action against clinically relevant stages, and, on the other hand, taking advantage of inhibiting the promastigote infective stage as a prophylactic approach.

One approach to improving anti-amastigote activity was advanced by Kückelhaus et al. [108] and relied on the hypothesis that the immune response triggered by the peptide’s interaction with the macrophage could amplify signals that affect the parasite’s survival. These authors evaluated phylloseptin–1 as an inhibitor of the progression of infection by *L. amazonensis* in macrophages and confirmed that the peptide reduced the number of infected macrophages and the amastigote load, while stimulating the production of signal molecules such as IL–12 and TNF–α, which seemed to enhance the macrophages’ ability to eliminate the parasites. Further studies within this line of action are fundamental to help in the understanding of these mechanisms, and such knowledge can be used to design new approaches to efficiently eliminate intracellular amastigotes.

On a different note, one must bear in mind that leishmaniasis is a vector-borne disease that, along with other such diseases like malaria or trypanosomiases, has given rise to the formulation of plans and strategies aimed at increasing early diagnosis, improving patient care and access to medicines, and reinforcing preventive measures focused on vector control. However, wild nature and ecological parameters make vector control a very difficult task, which further interferes with the effectiveness of other measures. Given the need to find better solutions for these vector-borne diseases, the WHO supports research on paratransgenesis as applied to the control of, e.g., leishmaniasis, malaria, Chagas disease, and yellow fever. Paratransgenesis is a useful methodology based on the modification of symbiotic or commensal microorganisms of vectors to produce molecules that inhibit parasitic transmission [109]. Support for this line of action is provided if the procedure is carried out through an approach validated by government statutes that address any ecological, environmental, and health impacts [110]. Although this is promising, paratransgenesis has several requirements for its correct implementation, such as the presence of a microorganism that can live under the internal conditions of the vector, which is cultivable, resistant to transformation processes, stable in its ability to produce the desired molecule, and non-pathogenic to mammalian hosts.

Amongst the many leishmanicidal peptides reported in the literature and summarized herein, several candidates could be devised as compatible to engaging into this process. However, it cannot be disregarded that such peptides should preferably be of short length, active at low concentrations, and not cause any damage to the producing microorganism. This is not easy to achieve, although promising results have already been reported for Chagas disease and malaria [111,112], bringing hope to the fight against leishmaniasis by similar means.

Additionally, in connection with vector control, a ‘flying vaccination’ strategy was presented by Yamamot and Yoshida [113] using a saliva protein from the sand fly *Phlebotomus papatasi* known as SP15. This molecule was selected because *P. papatasi*’s saliva increases *Leishmania* infection. Mice bitten with transformed mosquitoes presented high levels of anti-SP15 antibodies, confirming the stability and immunogenicity of the selected protein when transferred to the mammalian host. This study provided a proof of concept on aspects such as the correct transformation of the vectors, the expression and location of the introduced molecule, and its ability to generate an immune response in the individual. Although this is an important step that provided clues towards the advancement of the frontiers of knowledge in antileishmanial research, more studies are still needed to evaluate the functionality and universality of this vaccination strategy.

## 7. Concluding Remarks

Despite its overwhelming burden worldwide, leishmaniasis has attracted practically no interest from researchers aimed at developing adequate vaccines or drugs. In other words, although they are desperately needed, novel efficient antileishmanial therapies have often been neglected. However, both global warming and the increasing mobility of populations between countries and continents are expanding the typical subtropical distribution of this disease to include more temperate zones, where developed countries are located. Hopefully, this will change mentalities and trigger a paradigm shift in the fight against leishmaniasis and other NTDs.

Considering that in recent years peptides have been emerging as an important class of bioactive molecules with therapeutic potential against infectious diseases, including leishmaniasis, the present review is a timely one. We have gathered over 140 unique peptide sequences in a database, and a total of nearly 300 entries showing inhibitory action on different species of these parasites, some on both forms of life—promastigote and amastigote—were analyzed and compared. Such bioactive molecules possess different combinations of amino acids and of physicochemical parameters, which underpin their diverse effects, potency, and mechanisms of action. A thorough analysis of these relationships can provide important clues for the future development of therapies and strategies to control *Leishmania* spp.

Many aspects of this area of research remain untapped, such as the assessment of dual-stage antileishmanial action or of the in vivo efficacy of many of the reported peptides, strategies for the optimization of their in vivo performance, or their value in transgenesis- or paratransgenesis-based approaches towards the prevention of transmission. Computational models and tools for the prediction of antileishmanial peptide activity are also urgently needed. Future investment in these topics can contribute to increasing our knowledge in this field of research and provide highly useful tools for the design of better alternatives to tackle this long-standing and long-neglected health burden.

## Data Availability

The database elaborated and used for the writing of this paper can be downloaded from https://github.com/albert-robles1101/Leishmania-database, accessed on 25 March 2021.

## Supplementary Materials

The following are available online at https://www.mdpi.com/article/10.3390/ijms22094400/s1. Table S1: Exploratory summary of the physicochemical properties of leishmanicidal peptides.
