# Supplementary material for: Peptides to Tackle Leishmaniasis: Current Status and Future Directions
Source: Int J Mol Sci. 2021 Apr 22;22(9):4400. doi: 10.3390/ijms22094400 (PMC8122823; doi:10.3390/ijms22094400)
Supplement: Supplementary file 1 [file ijms-22-04400-s001.zip › ijms-1177933-supplementary.pdf]

Table 1. S: Exploratory summary of the physicochemical properties of leishmanicidal peptides.

	Aliphatic Index	Boman Index	Charge	Hydrophobic moment	Hydrophobic index	Instability index	Length	Molecular Weight	Isoelectric Point
$\underline{x}$	114.63	0.87	4.07	0.56	0.19	31.99	21.48	2412.66	10.58
ϑ	53.30	2.64	2.99	0.15	1.21	67.58	11.22	1227.57	1.75
**Min.**	0.00	-2.39	-9.27	0.15	-3.05	-36.48	4.00	503.55	3.79
**25%**	81.03	-0.95	2.09	0.49	-0.53	-0.09	13.00	1606.03	9.70
**50%**	120.00	0.16	3.09	0.52	0.39	17.53	19.00	2075.52	10.81
**75%**	146.25	2.31	5.94	0.66	1.18	41.77	27.00	3000.77	11.50
**Máx.**	232.31	10.69	12.87	0.95	2.02	353.11	84.00	9702.00	13.45
**Skew**	-0.25	1.63	-0.06	0.14	-0.78	3.08	1.98	2.07	-1.41
**Kurt.**	-0.42	3.41	2.82	0.20	0.08	10.50	6.50	7.04	3.51
